# Expression at the Imprinted Dlk1-Gtl2 Locus Is Regulated by Proneural Genes in the Developing Telencephalon

**DOI:** 10.1371/journal.pone.0048675

**Published:** 2012-11-06

**Authors:** Julie Seibt, Olivier Armant, Anne Le Digarcher, Diogo Castro, Vidya Ramesh, Laurent Journot, François Guillemot, Pierre Vanderhaeghen, Tristan Bouschet

**Affiliations:** 1 IRIBHM (Institute for Interdisciplinary Research), Université Libre de Bruxelles (ULB), Brussels, Belgium; 2 Neuroscience Research Center (NWFZ), Campus Charité Mitte, Berlin, Germany; 3 Division of Molecular Neurobiology, National Institute for Medical Research, The Ridgeway, Mill Hill, London, United Kingdom; 4 CNRS, UMR-5203, Institut de Génomique Fonctionnelle, Montpellier, France; 5 INSERM, U661, Montpellier, France; 6 Universités de Montpellier 1 & 2, UMR-5203, Montpellier, France; 7 Welbio, Université Libre de Bruxelles (ULB), Brussels, Belgium; King's College London, United Kingdom

## Abstract

Imprinting is an epigenetic mechanism that restrains the expression of about 100 genes to one allele depending on its parental origin. Several imprinted genes are implicated in neurodevelopmental brain disorders, such as autism, Angelman, and Prader-Willi syndromes. However, how expression of these imprinted genes is regulated during neural development is poorly understood. Here, using single and double KO animals for the transcription factors Neurogenin2 (Ngn2) and Achaete-scute homolog 1 (Ascl1), we found that the expression of a specific subset of imprinted genes is controlled by these proneural genes. Using in situ hybridization and quantitative PCR, we determined that five imprinted transcripts situated at the Dlk1-Gtl2 locus (Dlk1, Gtl2, Mirg, Rian, Rtl1) are upregulated in the dorsal telencephalon of Ngn2 KO mice. This suggests that Ngn2 influences the expression of the entire Dlk1-Gtl2 locus, independently of the parental origin of the transcripts. Interestingly 14 other imprinted genes situated at other imprinted loci were not affected by the loss of Ngn2. Finally, using Ngn2/Ascl1 double KO mice, we show that the upregulation of genes at the Dlk1-Gtl2 locus in Ngn2 KO animals requires a functional copy of Ascl1. Our data suggest a complex interplay between proneural genes in the developing forebrain that control the level of expression at the imprinted Dlk1-Gtl2 locus (but not of other imprinted genes). This raises the possibility that the transcripts of this selective locus participate in the biological effects of proneural genes in the developing telencephalon.

## Introduction

The cerebral cortex is populated by two main neuronal classes, pyramidal neurons and interneurons that represent respectively 85% and 15% of cortical neurons.

Two distinct, though tightly linked processes, control the development and placement of these neurons within the cortex. On one hand, ‘spatial patterning’ by which the telencephalon is regionally subdivided into defined morphological and molecular progenitor territories, thereby underlying the generation of future interneurons and pyramidal neurons [1], [2]. Hence pyramidal neurons and interneurons originate through two distinct embryonic structures, the dorsal and the ventral telencephalon respectively [1], [3]. On the other hand, the process of ‘temporal specification’ describes the capacity of cortical progenitors to evolve over time to produce different types of neurons and give rise to the typical laminar organization of the cortex [1], [4].

The proneural genes Ngn2 and Ascl1 are respectively expressed in the dorsal and ventral telencephalon, where they determine the regional identity of neural progenitors [5], [6]. Interestingly, in Ngn2 KO mice, dorsal progenitors are partially respecified towards a ventral phenotype as a result of Ascl1 ectopic expression in the presumptive cortex [5]. Moreover, Ngn2 and Ascl1 regulate temporal patterning [7], [8] and coordinate a wide range of basic cellular processes such as cell cycle exit, migration or neuronal connectivity [9], [10].

During recent years a series of transcriptional targets of Ngn2 and Ascl1 that could mediate their functions have been reported [11], [12], [13], [14]. However, the list of these molecular effectors is still far from completion.

Parental genomic imprinting is an epigenetic mechanism that restrains the expression of about 100 mammalian genes to one parental allele [15]. In other words, in contrast to the vast majority of genes that display biallellic expression, imprinted genes (IGs) are differentially transcribed depending on the parental origin of the allele; for example the imprinted *H19* gene is transcribed from the maternal allele only (maternally expressed gene; MEG), whereas its imprinted neighbor *Igf2* is a paternally expressed gene (PEG) [15]. The differential transcription depending on the parental origin of the allele is due to methylation marks that are selectively deposited on differentially methylated regions (DMRs) during male and female gametogenesis by specific *de novo* DNA methyltransferases (Dnmt) [15], [16].

Both MEGs and PEGs are required during mouse embryonic development as uniparental embryos (inheriting 2 copies of maternal or paternal genomes) that express only PEGs or only MEGs, die during early development [17], [18], [19]. Furthermore, mouse mutants for individual IGs display altered phenotypes, including embryonic growth restriction or overgrowth [15]. As IGs are transcribed from a single parental allele, the loss of the transcriptionally active allele results in a complete loss of gene expression while loss of imprinting results in an abnormal biallellic expression [15], [20], [21], [22]. There is growing experimental and clinical evidence that misregulated expression of IGs is involved in human pathologies affecting brain function [15], [21], [22]. At least one IG (*Ube3a*) is linked to Angelman disease and several imprinted loci are linked to Autism Spectrum Disorders (ASD) and to Prader-Willi, two syndromes in which cerebral functions are altered [21], [23]. Very recently the PEG Dlk1 was shown to be required for neurogenesis in the postnatal brain SVZ [24], a process that depends on Ascl1 [25], [26]. However, to date there is no reported link between imprinted genes expression and proneural genes.

Here we identify 5 imprinted genes, 2 PEGs (Dlk1, Rtl1) and 3 MEGs (Gtl2, Rian, Mirg) situated at an imprinted locus at 12qF1 [15] as potential targets of Ngn2 and Ascl1, raising the possibility that they participate in brain development.

## Results

**Figure 1:**
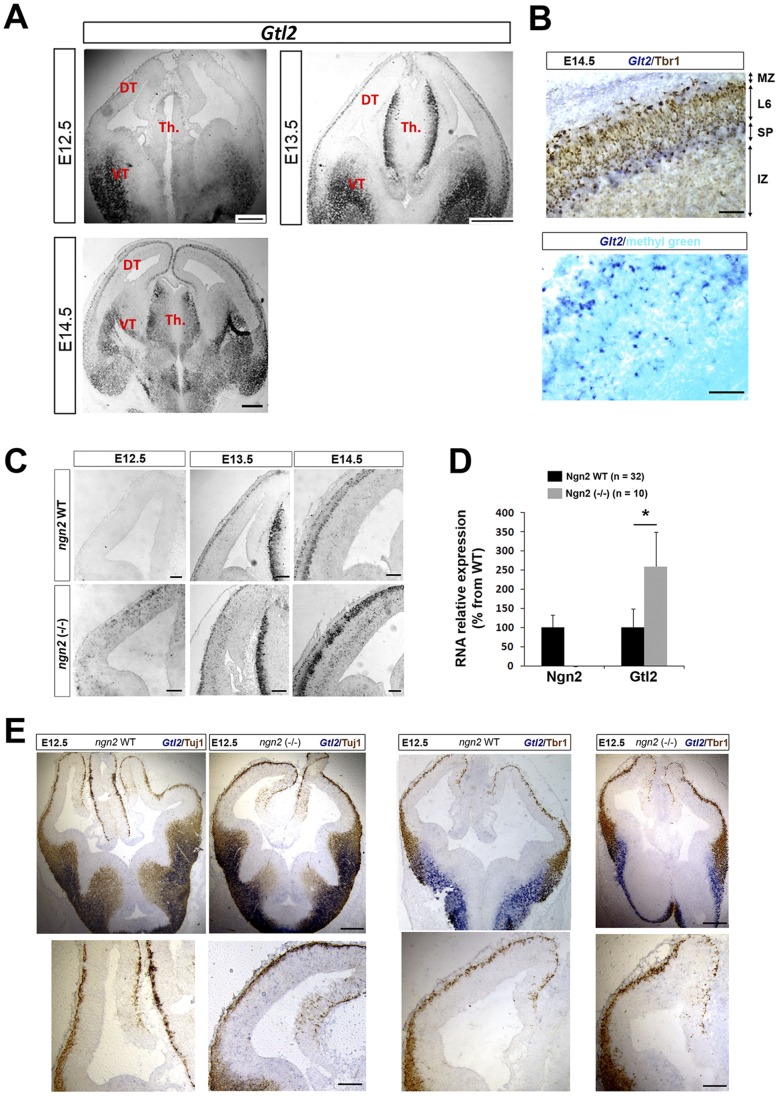
Gtl2 is upregulated and ectopically expressed in the dorsal telencephalon of Ngn2 KO mice. A. Detection of Gtl2 RNA at E12.5, E13.5 and E14.5 by ISH in WT mice. DT = dorsal telencephalon, VT = ventral telencephalon, Th. = thalamus. **B.** Expression of Gtl2-positive neurons in the subplate (SP) and marginal zone (MZ) but not in Tbr1 (layer 6) positive neurons of the developing cortical plate (upper panel). Gtl2 ISH counterstained with the nuclear marker methyl green in the cortex (lower panel). **C.** Gtl2 expression (ISH) in the dorsal telencephalon of WT and Ngn2 KO mice at E12.5, E13.5, and E14.5. **D.** Overexpression of Gtl2 in Ngn2 KO dorsal telencephalon at E13.5 assessed by qPCR. Data are presented as percentage of change normalized to the mean of WT (taken as 100%) + s.e.m; *P<0.01 in Mann-Whitney’s test. **E.** ISH of Gtl2 co-labeled with Tuj1 (4 left panels) and Tbr1 (4 right panels) proteins (immunohistochemistry) shows that Gtl2 is ectopically expressed in Tuj1 and Tbr1 negative regions in Ngn2 KO mice. Scale bars: A: 400 µm. B: 50 µm. C and E upper panels: 200 µm, C and E lower panels: 50 µm.

In a microarray screen to identify Ngn2 targets (JS, TB and PV, unpublished data), we observed that the non-coding RNA encoded by the imprinted gene *Gtl2* (Gene trap locus 2, also known as Maternally expressed gene 3) was highly upregulated in the dorsal telencephalon of Ngn2 KO mice. First, to shed light on Gtl2 localization and infer about its function in the normal developing telencephalon, we performed in situ hybridizations (ISH) on WT animals. At E12.5, Gtl2 is mostly expressed in postmitotic neurons of the ventral telencephalon where it remains strongly expressed up to E17.5 (**Fig.1A** and **Fig.S1**). From E13.5 onward, Gtl2 starts to be expressed in the nascent cortical plate in the dorsal telencephalon (**Fig.1A−C**), where it is expressed mostly in early generated neurons forming the marginal zone (MZ) and subplate (SP) at E14.5 (**Fig.1B**). Gtl2 RNA is also abundant in the neighboring thalamus (Fig.1A). As previously reported for the muscle [27], Gtl2 RNA is localized mostly in the nucleus in the developing forebrain (**Fig.1B**).

In addition, by using reciprocal hybrid cortex issued from crosses between *M. m. domesticus* and *M. m. molossinus*, we were able to distinguish between parental alleles and to determine that Gtl2 is maternally expressed in the developing dorsal telencephalon (T.B. and L.J., unpublished observation), confirming that Gtl2 is a MEG at this developmental stage in this structure. We then confirmed by ISH (**Fig.1C**) and quantitative PCR (qPCR) (**Fig.1D**) the upregulation of Gtl2 in Ngn2 KO cortex. In Ngn2 KO mice, we did not observe co-localization of Gtl2 RNA with the postmitotic neuronal markers Tuj1 and Tbr1, indicating that the cells expressing ectopically Gtl2 located in the ventricular and intermediate zones are likely progenitor cells (**Fig.1E** and **Fig.S1B,D**). We didn’t detect any changes in Gtl2 expression in the ventral telencephalon at any developmental stages in Ngn2 KO mice (data not shown). This data thus shows that in the developing telencephalon, the imprinted gene Gtl2 encodes for a nuclear RNA expressed mostly in post-mitotic neurons of the ventral and dorsal telencephalon and thalamus (**Fig.1A**). Upon loss of Ngn2, Gtl2 becomes ectopically and precociously expressed in the dorsal telencephalon. We also found that the cells overexpressing Gtl2 in Ngn2 KO are not yet differentiated (Tuj1 negative) at early embryonic stages (E12.5, **Fig.1E**) and located predominantly in the intermediate zone (**Fig. S1B, D**).

**Figure 2:**
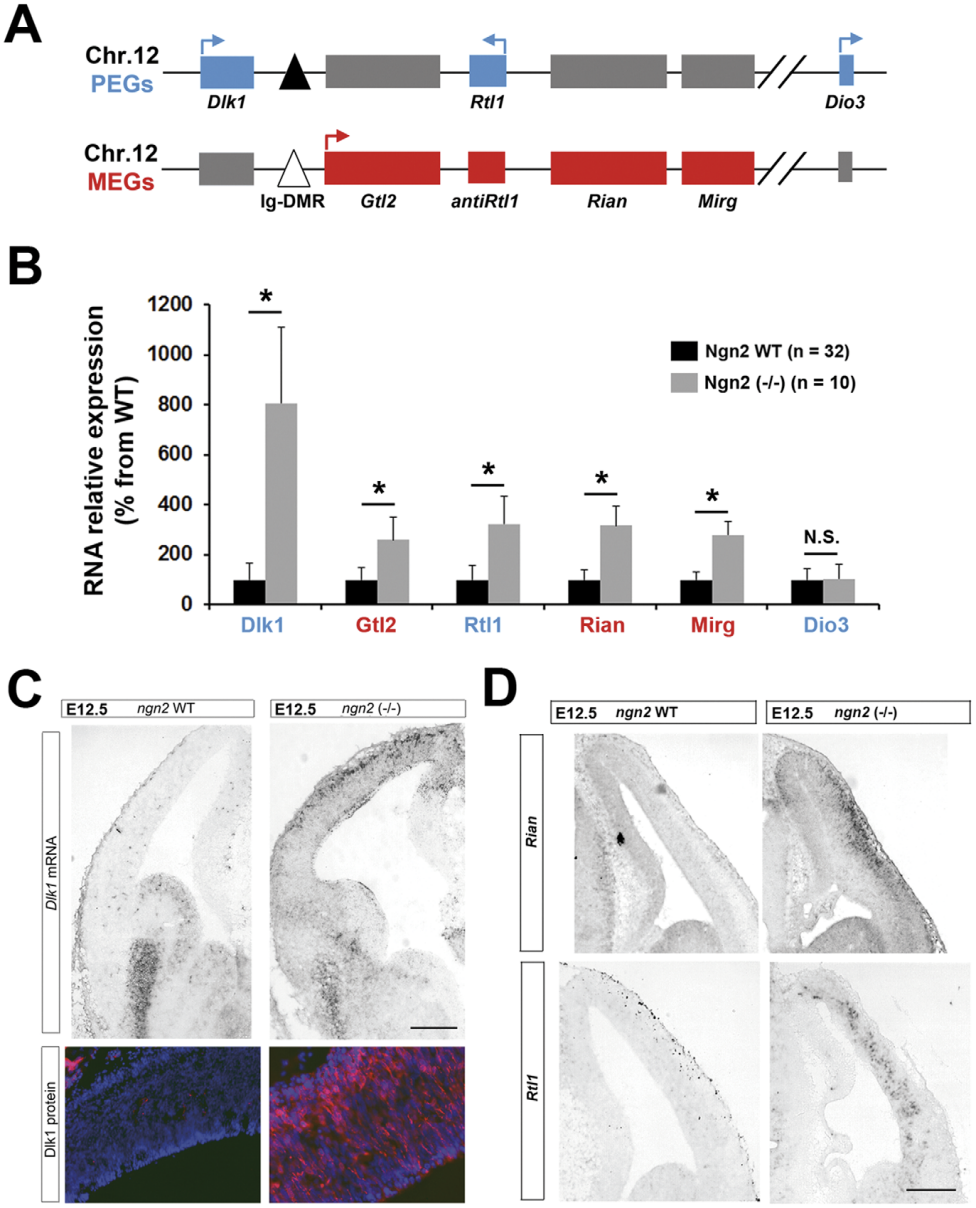
Upregulation and ectopic expression of imprinted genes at the Dlk1-Gtl2 locus in Ngn2 KO mice. A. Schematic representation of the Dlk1-Gtl2 locus. The active transcribed alleles are colored in blue for PEGs and red for MEGs. The silenced alleles are colored in grey. The control region Ig-DMR is represented by a triangle. Arrows indicate the sense of transcription. Adapted from [15]. **B.** qPCR analysis of RNAs expression for Dlk1, Gtl2, Rtl1, Rian, Mirg, and Dio3 in Ngn2 WT and Ngn2 KO DT at E13.5. Data are presented as percentage of change normalized to the mean of WT (taken as 100%) + s.e.m. *P<0.01**.** Transcription of Dio3 did not change significantly (NS, Mann-Whitney’s test). **C.** Dlk1 mRNA and protein expression assessed respectively by ISH (upper panels) and immunohistofluorescence (lower panels) in E12.5 Ngn2 WT and Ngn2 KO animals. **D.** Cortical RNA expression for Rian and Rtl1 in E12.5 WT and Ngn2 KO animals assessed by ISH. Scale bars in C and D: 200 µm.

Gtl2 is part of an imprinted locus at chromosomal position 12qF1 that contains several additional MEGs and PEGs including Dlk1, Rian (also known as Meg8), Mirg and Rtl1 (also known as Peg11) (**Fig.2A**) [15]. To determine whether the level of expression of these IGs is also affected in the cortex of Ngn2 KO mice, we performed qPCR on cDNA samples from Ngn2 WT and Ngn2 KO cortex at E13.5. As shown **in** **Figure 2B**, Dlk1, Gtl2, Rtl1, Rian, and Mirg were all significantly upregulated upon Ngn2 loss. By contrast Dio3, which is situated at the extremity of the locus (**Fig.2A**), was unaffected. We confirmed by ISH that expression of Dlk1, Rian and Rtl1 are upregulated and that they are ectopically expressed in Ngn2 KO cortex (**Fig.2C,D**). We also observed by immunohistofluorescence an increase in the protein product of *Dlk1* gene in Ngn2 KO cortex (**Fig.2C**), an increase that parallels those of RNA. These data thus show that 5 IGs of the Dlk1-Gtl2 locus are upregulated in the cortex of Ngn2 KO mice. This upregulation seems to be independent of the parental origin of the transcripts as on these 5 IGs, 3 are established MEGs (Gtl2, Mirg, Rian) while 2 are PEGs (Dlk1, Rtl1) (**Fig.2A**).

**Figure 3:**
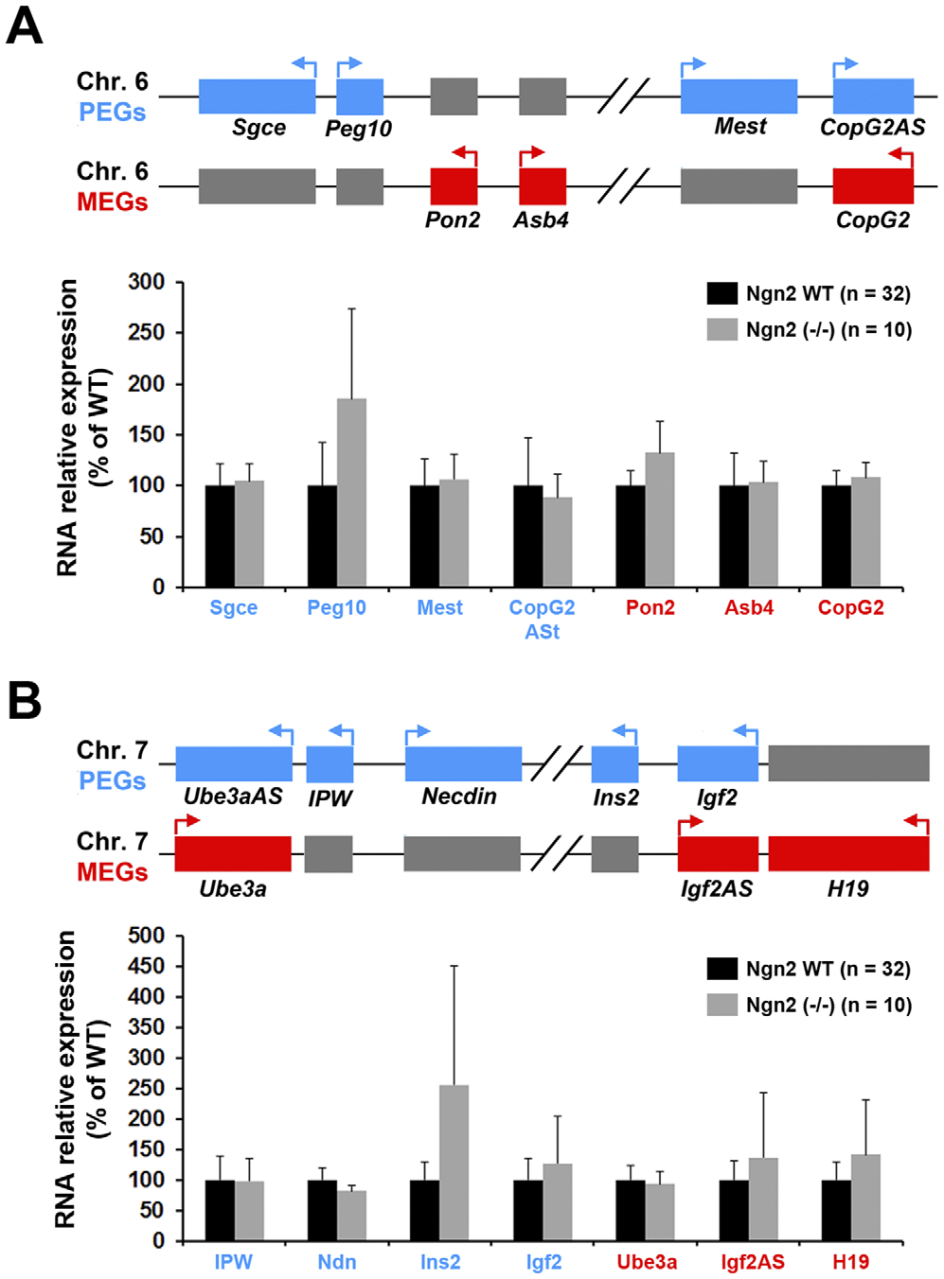
Expression of imprinted genes outside the Dlk1-Gtl2 locus is not affected in Ngn2 KO mice. **A.** Changes in RNA expression assessed by qPCR in Ngn2 WT and KO mice of 7 IGs on chromosome (Chr.) 6. A schematic representation of the locus is shown above the graphs. **B.** Changes in RNA expression assessed by qPCR in Ngn2 WT and KO mice of 7 IGs on Chr.7. A schematic representation of the locus is shown above the graphs. For both loci, the active transcribed alleles are colored in blue for PEGs and red for MEGs. The silenced alleles are colored in grey. Arrows indicate the sense of transcription. Expression of none of the 14 IGs was significantly affected in Ngn2 KO mice (Mann-Whitney’s test).

To determine whether the absence of Ngn2 selectively affects the Dlk1-Gtl2 locus or whether it also impacts on IGs located at other genomic regions, we measured RNA levels of 14 additional IGs present on 4 imprinted loci. These loci are situated at chromosomes 6 (**Fig.3A**) and 7 (**Fig.3B**). They were selected because they either contain IG involved in brain development (Necdin [28], Mest [29], Igf2 [30]) or because they contain IGs implicated in neurodevelopmental diseases (Angelman: Ube3A, Prader-Willi: Necdin, IPW). These 14 genes therefore represent potential targets for factors involved in brain development such as Ngn2 and Ascl1. Strikingly, as shown in **Figure 3**, none of these 14 IGs was significantly affected in the presumptive cortex of Ngn2 KO mice. This indicates that loss of Ngn2 in the cortex likely selectively impacts expression on IGs situated at the Dlk1-Gtl2 locus.

**Figure 4:**
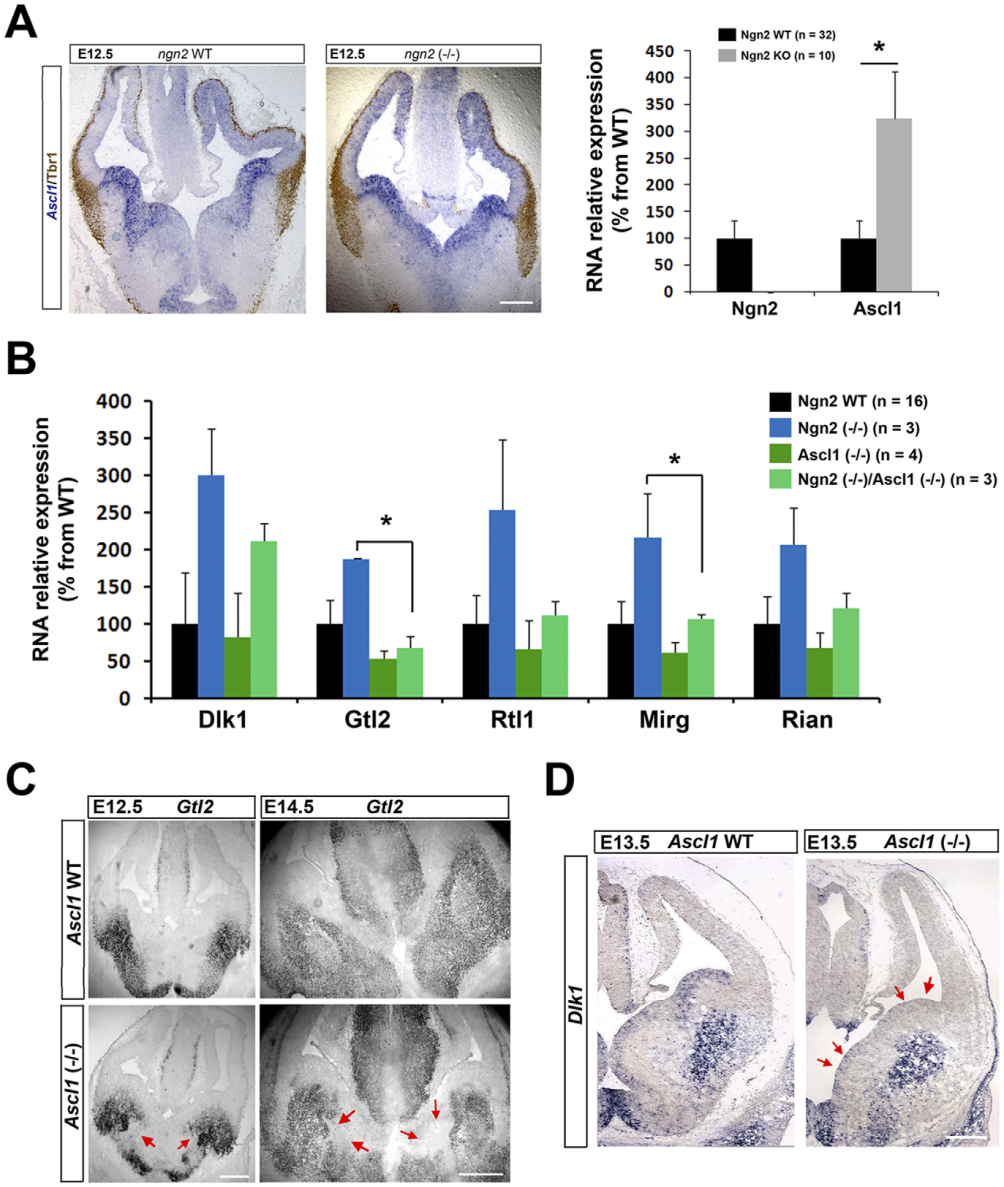
Role of Ascl1 in the upregulation of genes of the Dlk1-Gtl2 locus in Ngn2 KO mice. A. Upregulation of Ascl1 mRNA in the dorsal telencephalon from WT or Ngn2 KO was determined by ISH (E12.5, left panel) and qPCR experiments (E13.5, right panel). **B.** Expression of imprinted RNAs from the Gtl2-Dlk1 locus (Dlk1, Gtl2, Rtl1, Mirg, Rian) in WT, Ngn2 KO, Ascl1 KO and Ngn2/Ascl1 double KO in E13.5 dorsal telencephalons. Data are presented as percentage of change normalized to the mean of WT (taken as 100%) + s.e.m; *P<0.01 in Mann-Whitney’s test. **C.** RNA expression (ISH) of Gtl2 in WT and Ascl1 KO mice at E12.5 and E14.5. **D.** RNA expression (ISH) of DLK1 in WT and Ascl1 KO mice at E13.5. Red arrows in C and D indicate regions in the ventral telencephalon where expression of Gtl2 and DLK1 are reduced in Ascl1 KO mice. Scale bars: A,B,D = 200 µm, C = 400 µm.

We next thought to identify the molecular mechanism responsible for the overexpression of Dlk1, Gtl2, Rian, Mirg and Rtl1 in the Ngn2 KO dorsal telencephalon. One obvious candidate to test is Ascl1 that is known to be highly upregulated in the cortex of Ngn2 KO animals (**Fig.4A** and [5]). Furthermore Ascl1 ectopic expression in the Ngn2 positive territory is sufficient to respecify the dorsal telencephalon towards a ventral phenotype [5]. To determine whether Ascl1 is required for upregulation of transcripts at the Dlk1-Gtl2 locus, we compared the expression of Dlk1, Gtl2, Rian, Mirg and Rtl1 in the dorsal telencephalon of WT, Ngn2 KO, Ascl1 KO and Ngn2/Ascl1 double KO mice. The observed upregulation of IGs in the dorsal telencephalon of Ngn2 KO mice was counteracted by the absence of a functional copy of Ascl1 in Ngn2/Ascl1 double KO mice as illustrated by an average expression of RNAs for Dlk1, Gtl2, Mirg, Rtl1 and Rian that was lower in double KO mice compared to single Ngn2 KO mice (**Fig.4B**). The values for Dlk1, Rian and Rtl1 were not significantly different between Ngn2 KO and Ngn2/Ascl1 double KO mice (Mann-Whitney test). We attribute this to variations within the simple and double KO categories and to the small proportion of animals in each of these categories, which is principally due to the fact that double KO animals are difficult to obtain. Nevertheless, values for Gtl2 and Mirg were significantly different between Ngn2 KO and Ngn2/Ascl1 double KO mice, indicating that at least 2 genes of the locus are unambiguously affected by the absence of Ascl1**.** To further investigate the link between Ascl1 and IGs at the Dlk1-Gtl2 locus, we performed ISH on Ascl1 KO mice. We confirmed that Ascl1 is directly linked to the expression of at least two imprinted transcripts (Gtl2 and Dlk1) in the ventral telencephalon. As shown in **Figure 1A** and **Figure** **2C**, both IG transcripts are expressed in the ventral telencephalon in WT animals where Ascl1 is normally expressed, but their RNA expression in this territory is notably attenuated in Ascl1 KO mice (**Fig.4C,D**). These results confirms that Ascl1 regulates the expression of both IGs and reinforces our hypothesis that upregulation of Ascl1 in Ngn2 KO mice in the dorsal telencephalon is responsible for ectopic expression of those genes in this territory.

Altogether, this suggests a model in which Ascl1 plays a central role in regulating the expression of IGs of the Dlk1-Gtl2 locus in the developing brain. First, Ascl1 would be necessary for their expression in the normal developing ventral telencephalon. Secondly, the upregulation of the RNAs from the Dlk1-Gtl2 locus in Ngn2 KO animals would be a consequence of the ectopic expression of Ascl1 in the respecified dorsal telencephalon.

## Discussion

This study has uncovered a link between neurogenic transcription factors and a set of imprinted transcripts. First, we have observed that the imprinted gene Gtl2 is upregulated in the telencephalon of Ngn2 KO animals (**Fig.1C, D**). Gtl2 is an imprinted gene of maternal expression (hence its name; Meg3) that is highly expressed during embryogenesis [27], notably during brain development. Confirming these observations, we have shown that Gtl2 is first abundantly expressed in the ventral telencephalon and later on at weaker levels in the dorsal telencephalon (**Fig.1**). Gtl2 is crucial for mouse development as its inactivation causes perinatal death and skeletal muscle defects [**31**], [**32**]. The mechanisms of action of this non-coding RNA are largely unknown but it was recently shown that there is an increased expression of angiogenic genes in the brain of Gtl2 KO mice [33].

Gtl2 is part of the Dlk1-Gtl2 imprinted locus at chromosomal position 12qF1 that contains several additional MEGs and PEGs [15]. The expression of these IGs is regulated by an imprinted center, the IG-DMR, which is differentially methylated during male and female gametogenesis [15]. Gtl2 deletion affects the methylation of the IG-DMR, yielding to a transcriptional silencing of MEGs while an enhancement of neighboring PEGs situated at 12qF1 [32]. Therefore, Gtl2 itself participates in controlling the expression at its own locus.

Consistent with the notion of coregulation, we show here that the level of transcripts for Gtl2 but also those of Dlk1, Mirg, Rian, Rtl1, are all increased in Ngn2 KO mice (**Fig.2**). To our knowledge, this is the first case report of a situation where all IGs of the Dlk1-Gtl2 locus, independently of their parental origin, are upregulated. Interestingly, a mirror situation, but showing a common downregulation, was recently reported. Fibroblasts that are poorly prone to be reprogrammed during the process of generating induced pluripotent stem cells have an aberrant low level of genes situated at this locus [34]. As other IGs are not markedly affected by Ngn2 deletion in the forebrain (**Fig.3**), this suggests that proneural genes may specifically target this imprinted locus. This raises the question of what would be the advantage for a proneural gene to target an entire imprinted locus. A possibility would be that, these IGs are not capable to exert a function during development as individual genes but rather need to work in synergy. Few data are available to evaluate this hypothesis. One supporting set of data concerns the Dlk1 gene. Indeed, Dlk1 KO animals show normal embryonic neurogenesis but impaired postnatal SVZ neurogenesis [24], indicating that the loss of this single gene has no impact during prenatal development. A synergy between IGs could take place as meta-analysis of microarray data support the existence of a network of IGs [35]. Supporting this synergistic view, Gtl2 and Dlk1 KO mice have similar growth retardation phenotype and Gtl2 and Dlk1 are frequently co-expressed. Here we show that both transcripts are expressed in the ventral forebrain in WT mice and ectopically expressed in the dorsal telencephalon as a result of Ngn2 deletion. These normal and aberrant expressions likely rely on Ascl1 expression. Indeed, on one hand IGs are principally expressed in the Ascl1 positive territory, the ventral telencephalon (**Figs.1** **,** **4** and **Fig.S1**); on the other hand their upregulation in Ngn2 KO animals is reduced in Ngn2/Ascl1 double KO animals (**Fig.4**).

Each structure in the developing brain results from the coordinated action of specific combinations of transcriptions factors. In the dorsal telencephalon, Ngn2 and the master control gene Pax6 are co-expressed and are both necessary to complete corticogenesis [1]. By scrutinizing the list of genes differentially expressed between WT and Pax6 KO mice [36], we noticed that Pax6 deletion leads to an overexpression of Gtl2, Dlk1, Rian and of Ascl1. This set of genes is very similar to the one described here with Ngn2 KO mice. The fact that Ngn2 and Pax6 act on the same targets could therefore be a way to reinforce their combined action on patterning and specification. One possible explanation is that Pax6 and Ngn2 control IGs expression indirectly through the joint repression of Ascl1.

Finally, it would be exciting in the future to determine the identity of cells ectopically expressing IGs in Ngn2 KO mice and to perform gain and loss of function experiments where the levels of multiple genes from the Dlk1-Gtl2 locus are manipulated in order to investigate their impact on brain development.

## Materials and Methods

### Maintenance and Genotyping of *Ngn2^KIGFP^* and *Ascl1* Mutant Mice

The *Ngn2^KIGFP^* transgenic mice where eGFP has been inserted into the endogenous *Ngn2* locus by homologous recombination in ES cells have been described in [10]. The *Ascl1* transgenic mice have been described in [37]. To generate WT, heterozygous and homozygous (KO) Ngn2 animals, heterozygous *Ngn2^KIGFP^+/−* mice were intercrossed. To generate WT, Ascl1 KO, Ngn2 KO and Ascl1/Ngn2 double KO mice, *Ngn2^KIGFP^+/−; Asc1+/−* mice were intercrossed. The morning of vaginal plug detection is considered as the morning of the first day of gestation (E0.5). For analysis, WT and heterozygous animal were pooled as loss on one allele does not markedly alter phenotype [5], [6], [11]. Animal care and procedures were in compliance with local Ethics Committees (Université Libre de Bruxelles and Belgian National Fund for Scientific Research FRS/FNRS) and institutional guidelines. The ‘commission du bien être et de l’éthique animale’ (Welfare and animal ethics) of Faculty of Medicine, University of Brussels specifically approved this study (protocol number 259N).

### RNA Isolation and qRT-PCR

E13.5 dorsal telencephalons were dissected out from embryos obtained from *Ngn2ki^GFP^+/−* or *Ngn2ki^GFP^+/− Ascl1+/−* heterozygous intercrosses. RNA was isolated using RNeasy minikit according to the manufacturer’s instructions (Qiagen). Alternatively, RNA was extracted using RNA now (Ozyme). All RNA samples were treated with DNase (Qiagen or Ambion for RNeasy and RNA now methods respectively). Reverse transcription was performed using N6 primers and MMLV-RT (Promega). Quantitative PCR (qPCR) was performed in duplicate in 384 well plates using 2× SybrGreen Mix and a LC480 Real-Time PCR System (Roche). Results are presented as linearized Cp-values normalized to housekeeping genes TBP, Gus2 and Gapdh and the indicated reference value (2^-ΔΔCt^). The sequence of primers is available upon request.

### RNA In Situ Hybridization

All steps and solutions for RNA in situ hybridization (ISH) were achieved in RNase free environment. Embryos were perfused intracardiacally with a solution of 4% paraformaldehyde (in PBS) and the brains were post-fixed in the same fixative over night. Brains were then cryoprotected in 30% sucrose (in PBS). ISH was carried out on 10 µm cryostat sections (coronal cut) using digoxygenin-labeled (Roche) RNA probes as previously described [38]. We have generated probes antisense to RNAs for Gtl2, Rian, Dkl1, Mirg and Rlt1 using embryonic brain cDNA as templates. Sense probes were used as controls and generated no staining (not shown). The sequences of primers are available upon request. For co-labeling of ISH with Tuj1 (1/500, Covance) and Tbr1 (1/1000, a gift from Robert Hevner), revelation of the anti-digoxygenin antibody with NBT/BCIP was directly followed by several PBS washes and 10 min post-fixation in 4% paraformaldehyde. To unmask epitopes, slides were treated with 100% EtOH/0.3% hydrogen peroxide for 30 min at RT. This procedure was followed by standard protocol for DAB immunohistochemistry protocol. Counterstaining in 0.5% green methyl (in distilled water) was performed after NBT/BCIP revelation by application of the nuclear staining solution for 5 min at RT followed by immersion in distilled water to stop the reaction.

### Immunofluorescence Staining

Immunostaining was performed on 20 µm thick cryosections. Blocking solution consisted of PBS supplemented with 5% horse serum (Invitrogen), 0.3% Triton X-100 (Sigma) and 3% Bovine Serum Albumin (BSA; Sigma). Antibody solution consisted of PBS supplemented with 1% horse serum, 0.1% Triton X-100 and 3% BSA. Dlk1 or Tbr1 antibodies (a kind gift of Charlotte Harken Jensen and Robert Hevner respectively) were incubated overnight at 4°C and secondary during 2 hours at RT. Nuclei were stained with bisbenzimide (Hoechst#33258; Sigma). Sections were mounted with glycergel (DAKO).

### Imaging

Pictures of the *in-situ* RNA hybridization and immunofluorescence staining were acquired with an Axioplan2 Zeiss microscope and a Spot RT camera, converted in false colors and overlayed using Adobe Photoshop software.

### Statistical Analysis

Data are presented as mean + standard error of the mean of at least three biologically independent experiments or of WT and KO embryos. A Mann-Whitney’s test was used for comparing the distribution of data of measurements of RT-qPCR experiments.

## Supporting Information

Figure S1 **Expression of Gtl2 RNA (ISH) at E17.5 in Ngn2 WT and Ngn2 KO mice.** (**A-E**) Co-labeling with Tbr1 protein (immunohistochemistry) was used to show specific localization of Gtl2 positive neurons in the dorsal telencephalon (VZ = ventricular zone, IZ = intermediate zone, CP = cortical plate). Cells that show ectopic expression of Gtl2 mRNA in Ngn2 KO mice are localized in the IZ (red arrows in **B** and **D**). Scale bars: 150 µm.
(TIF)
