# Supplementary figures and images for: Expression at the Imprinted Dlk1-Gtl2 Locus Is Regulated by Proneural Genes in the Developing Telencephalon
Source: PLoS One. 2012 Nov 6;7(11):e48675. doi: 10.1371/journal.pone.0048675 (PMC3490856; doi:10.1371/journal.pone.0048675)

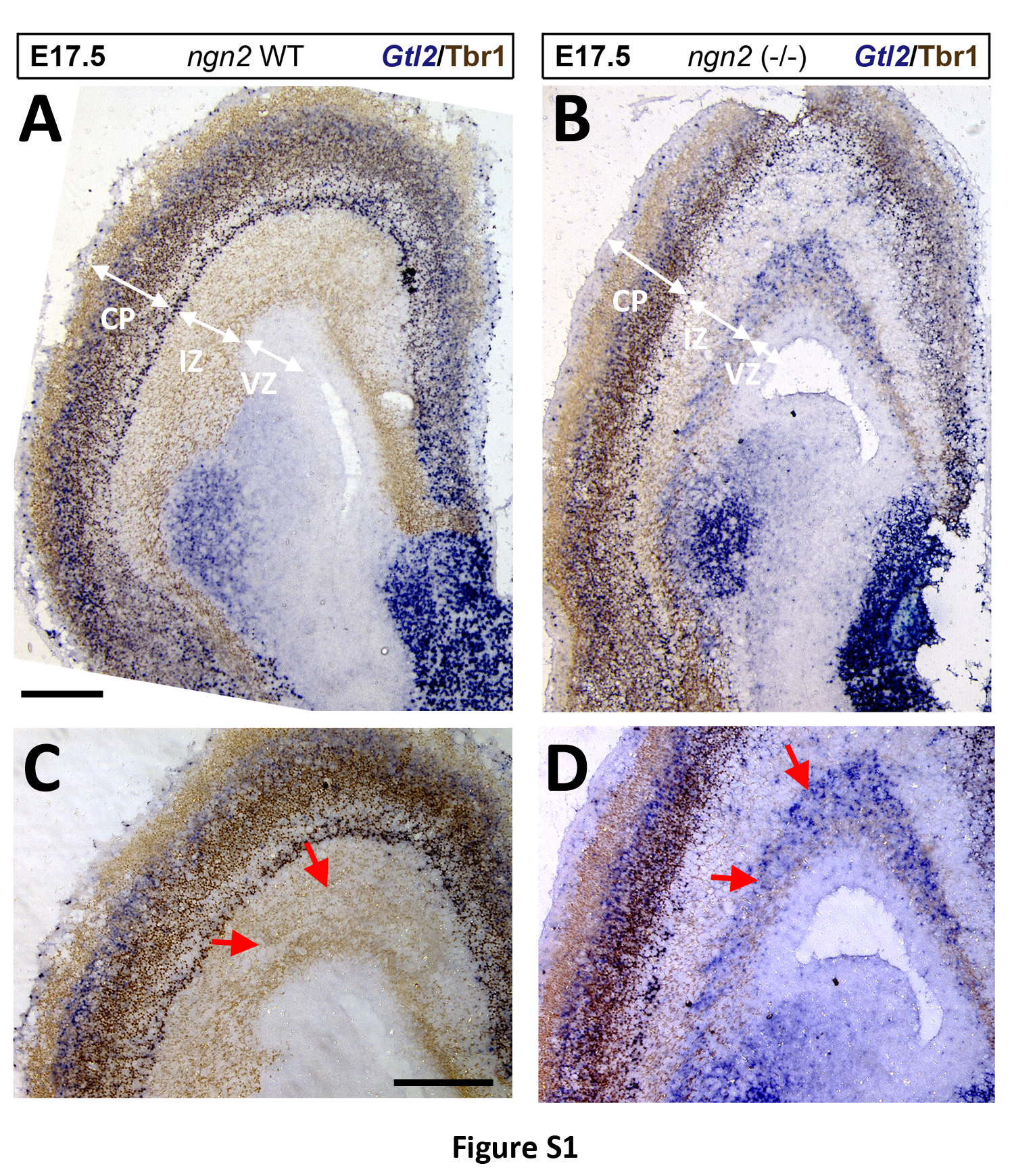

Supplement: Figure S1 — Expression of Gtl2 RNA (ISH) at E17.5 in Ngn2 WT and Ngn2 KO mice. (A-E) Co-labeling with Tbr1 protein (immunohistochemistry) was used to show specific localization of Gtl2 positive neurons in the dorsal telencephalon (VZ = ventricular zone, IZ = intermediate zone, CP = cortical plate). Cells that show ectopic expression of Gtl2 mRNA in Ngn2 KO mice are localized in the IZ (red arrows in B and D). Scale bars: 150 µm. (TIF) [file pone.0048675.s001.tif]
